# Insight into the molecular mechanisms of leaf coloration in *Cymbidium ensifolium*

**DOI:** 10.3389/fgene.2022.923082

**Published:** 2022-08-12

**Authors:** Hua Cao, Han Li, Xiang Chen, Yuying Zhang, Lin Lu, Shenchong Li, Xiang Tao, WeiYin Zhu, Jihua Wang, Lulin Ma

**Affiliations:** ^1^ Flower Research Institute Yunnan Agriculture Academy Science, Kunming, China; ^2^ Fujian Forestry Science and Technology Experimental Center, Zhangzhou, China; ^3^ Yunnan Agricultural University College of Horticulture and Landscape, Kunming, China; ^4^ Yunnan Agriculture Academy Science, Kunming, China

**Keywords:** leaf coloration, RNA-seq, flavonoid biosynthesis, chlorophyll accumulation, pigment

## Abstract

*Cymbidium* *ensifolium* L. is a significant ornamental plant in Orchidaceae. Aside from its attractive flowers, its leaf coloration is also an important ornamental trait. However, there is an apparent lack of studies concerning the intricate mechanism of leaf coloration in *C. ensifolium*. In this study, we report a systematic evaluation of leaf coloration utilizing transcriptome and metabolome profiles of purple, yellow, and green leaves. In total, 40 anthocyanins and 67 flavonoids were quantified along with chlorophyll content. The tissue–transcriptome profile identified 26,499 differentially expressed genes (DEGs). The highest chlorophyll contents were identified in green leaves, followed by yellow and purple leaves. We identified key anthocyanins and flavonoids associated with leaf coloration, including cyanidin-3-O-sophoroside, naringenin-7-O-glucoside, delphinidin, cyanidin, petunidin, and quercetin, diosmetin, sinensetin, and naringenin chalcone. Moreover, genes encoding UDP-glucoronosyl, UDP-glucosyl transferase, chalcone synthesis, flavodoxin, cytochrome P450, and AMP-binding enzyme were identified as key structural genes affecting leaf coloration in *C. ensifolium.* In summary, copigmentation resulting from several key metabolites modulated by structural genes was identified as governing leaf coloration in *C. ensifolium*. Further functional verification of the identified DEGs and co-accumulation of metabolites can provide a tool to modify leaf color and improve the aesthetic value of *C. ensifolium*.

## Introduction

*Cymbidium ensifolium* L., also known as sword-leaf cymbidium, is a perennial herbaceous plant belonging to Orchidaceae with its natural distribution across tropical and subtropical areas, including China, Indochina, Borneo, New Guinea, and the Philippines (Wang et al., 2011). *C. ensifolium* is an economically important ornamental with historical use in traditional Chinese orchids (Wang et al., 2011; Ai et al., 2021). Owing to its widespread commercial utilization in the horticulture industry and natural variation, several breeding programs have bred cultivars with distinct characteristics, including leaf blade, flower color, and morphology (Wang et al., 2011). Over the past few decades, many studies have focused on flower color variation in *C. ensifolium* (Choi et al., 2006; Siripiyasing et al., 2012; Li et al., 2014; Ogura-Tsujita et al., 2014; Nakatsuka et al., 2019; Ramya et al., 2020; Wei et al., 2020; Ke et al., 2021). However, the gradual change in leaf coloration is still not fully understood.

Although molecular mechanisms underlying leaf coloration are not fully understood in *C. ensifolium*, the conserved regulatory networks identified in other horticulture plants can provide a fundamental framework for understanding the molecular and genetic patterns underlying complex traits (Albert et al., 2014). Reports that are previously published suggested varying levels of co-accumulation of secondary metabolites such as betalains, flavonoids, and carotenoids responsible for plant pigmentations (Tanaka et al., 1998; Freyre and Griesbach, 2004; Grotewold, 2006; Yamamizo et al., 2011; Harborne, 2014). Flavonoids are involved in the formation and development of flowers, fruits, and seeds in plants and complement antioxidant activity, UV protection, and protection against biotic and abiotic attacks by plant pathogens (Dixon and Paiva, 1995; Winkel-Shirley, 2002). Quercetin and gossypetin are known flavonoids involved in synthesizing yellow pigments in plants (Harborne, 1965; Harborne, 1968; Ververidis et al., 2007; Li et al., 2020; Yin et al., 2020; Fu et al., 2021). Aside from pigmentation, flavonoids are involved in various physiological functions under stress conditions (Nakabayashi et al., 2014; Santos-Buelga et al., 2014; Fan et al., 2016; Liang and He, 2018; Xu and Rothstein, 2018). Phenylpropanoid biosynthesis, flavonoid metabolism, and anthocyanin metabolism are three major stages of flavonoid biosynthesis in plants. Flavonoid biosynthesis is aided by the structural genes *PAL*, *C4H*, *4CL*, *CHI*, *CHS*, *F3H*, *DFR*, *ANS*, *UFGT*, and *3GT* (Hichri et al., 2011).

With spectra of flower color, cymbidium is an integral part of flowering orchids in China (Xu et al., 2006; Aceto and Gaudio, 2011). Aside from abundance variation in flower color, leaf sheath and color are also important ornamental traits. A single plant displays varying colored leaves (at different developmental stages), which increases the aesthetic value of the species. Chlorophyll is the major pigment responsible for the green color in plants. Spatial regulation of anthocyanin and chlorophyll degradation are the primary sources of leaf color variation (Li et al., 2021). In general, color variation from white to yellow is attributed to the loss of chlorophyll, while red, purple, and blue coloration results from complex metabolic accumulation in flavonoid biosynthesis pathways (Wheeler and Smith, 2019). The previous report suggested that phenotypic mutation resulting from chlorophyll deficiency is the major reason for leaf color variation in *C. ensifolium* (Ai et al., 2021)*.* Moreover, the study demonstrated that the lower expression of photosynthesis–antennae caused white and yellow leaves (Ai et al., 2021). Another study concerning *C. ensifolium* also emphasized chlorophyll degradation as a major contributor to leaf coloration (Gao et al., 2020). However, the genetic mechanisms underlying leaf color variation of *C. ensifolium* from yellow–purple–green remain unclear. Therefore, the current study aimed at deciphering the gene expression change during leaf color change in *C. ensifolium* by utilizing a multi-omics platform with systematic analyses concerning high-quality transcriptome and metabolome profiles. The study will provide a theoretical basis for leaf coloration in *C. ensifolium*. Moreover, further functional validation of identified genes can establish the genetic basis of leaf coloration in *C. ensifolium.*

## Materials and methods

### Plant material

*Cymbidium ensifolium* var. “Shi zhang hong” is a popular orchid in the Chinese market. The 3-year-old plants were obtained from the orchid germplasm nursery in the Fujian Forestry Science and Technology Experimental Center (China), grown in the small rotted pine woods with an 80% air humidity, a 25°C temperature, and an 80% shading. The material was characterized by leaf color variation during the developmental process and a single plant displays varying colored leaves. The samples of purple (Z; young), yellow (H; slightly mature), and green (L; mature) were collected from a plant at the same time. Sampling was conducted in three biological replicates (three different plants) for further downstream analyses.

### Chlorophyll profiling

A series of procedures for chlorophyll profiling and other metabolites identification and quantification were carried out at Wuhan Metware Biotechnology Co., Ltd (https://www.metware.cn), following the company’s standard procedures. The chlorophyll a and b were extracted by hexane:acetone:ethanol = 2:1:1. The other metabolites such as porphobilinogen, hemin, pheophorbide a, protoporphyrin IX, 5-aminolevulinic acid, and L-glutamic acid were extracted using 70% methanol. Extracts were analyzed using liquid chromatography–mass spectrometry (LC-MS) analysis. After using the Metware Database constructed from the standards, the data detected *via* mass spectrometry are qualitatively and quantitatively analyzed.

### Measurement of total anthocyanin content

The method of extracting total anthocyanins from *Camellia sinensis* by Fu et al. (2021) was used, and the extraction conditions were optimized. *C. ensifolium* leaves were ground into powder in liquid nitrogen. About 0.65 g of dry powder was added to 20 ml of 95% (0.1 mol^−1^ HCL) ethanol solution and then heated in a water bath at 60°C for 2 h. At last, the absorbance values of the extracts at 520, 620, and 650 nm were measured using an enzyme marker, and 95% ethanol (0.1 mol^−1^ HCL) was used as A = (A530-A620)-0.1 (A650-A620), Q = A × V× 1,000/489.72 m (mmol.g^−1^ FW), where V represents the volume of the extract and m represents the weight of the dried petal powder.

### Flavonoids identification and quantification

A series of procedures for metabolite extraction, identification, and quantification were carried out at Wuhan Metware Biotechnology Co., Ltd (https://www.metware.cn), following the company’s standard procedures (Cao et al., 2019; Wang et al., 2019). Cryo-preserved samples were weighed and extracted with 1.0 ml of 70% methanol at 4°C. Extracts were analyzed using LC-MS/MS analysis (UPLC, Shim-pack UFLC SHIMADZU CBM30A system; MS, Applied Biosystems 6500 QTRAP). All metabolites were identified and quantified using Metware’s metabolite database and public metabolite database. Differentially accumulated flavonoids (DAFs) between samples were identified using orthogonal partial least squares discriminant analysis. Metabolites with |log_2_Fold Change| ≥ 1 and variable importance in project ≥ 1 were defined as differentially accumulated metabolites.

### RNA extraction and sequencing transcriptome data analysis

Total RNA was extracted from the samples with the RNA Extraction kit (TIANGEN, Beijing, China). The RNA quality and concentration were assessed using agarose gel electrophoresis and NanoDrop2000 spectrophotometer. RNA sample quality testing, library construction, and sequencing for each sample were done at Biomarker Biotechnology (http://www.biomarker.com.cn), following the company’s standard procedures. Low-quality data containing adapter and poly-N were removed for downstream analysis. The resulting set of high-quality clean reads was used for transcriptome analysis. Hisat2 was used to obtain unigenes (Kim et al., 2019). Reads per kilobase mapping (FPKM) for all genes to determine gene expression values and screen for differentially expressed genes (DEGs). DESeq2 (Love et al., 2014) was used for differential expression analysis between sample groups. DESeq2 requires unsorted reads count data for the input gene, not RPKM, FPKM, etc. After the difference analysis, the Benjamini–Hochberg method was used to correct the hypothesis test probability (*p-*value) for multiple hypothesis testing to obtain the false discovery rate (FDR). The filter for differential genes is |log_2_Fold Change| > = 1 and the FDR < 0.05. The identified DEGs were further enriched using the Kyoto Encyclopedia of Genes and Genomes (KEGG) analysis. The Gene Ontology annotation and KEGG pathway enrichment analysis were applied using Tbtools software (Chen et al., 2020). Heat maps were generated using the OmicStudio tools at https://www.omicstudio.cn/tool. Correlation analysis between key genes in the phenylpropanoid–flavonoid pathway and anthocyanins was performed using the R package Hmisc (Team, 2013) to calculate Pearson correlation coefficients.

## Results

### Phenotypic characterization of leaf coloration

**FIGURE 1:**
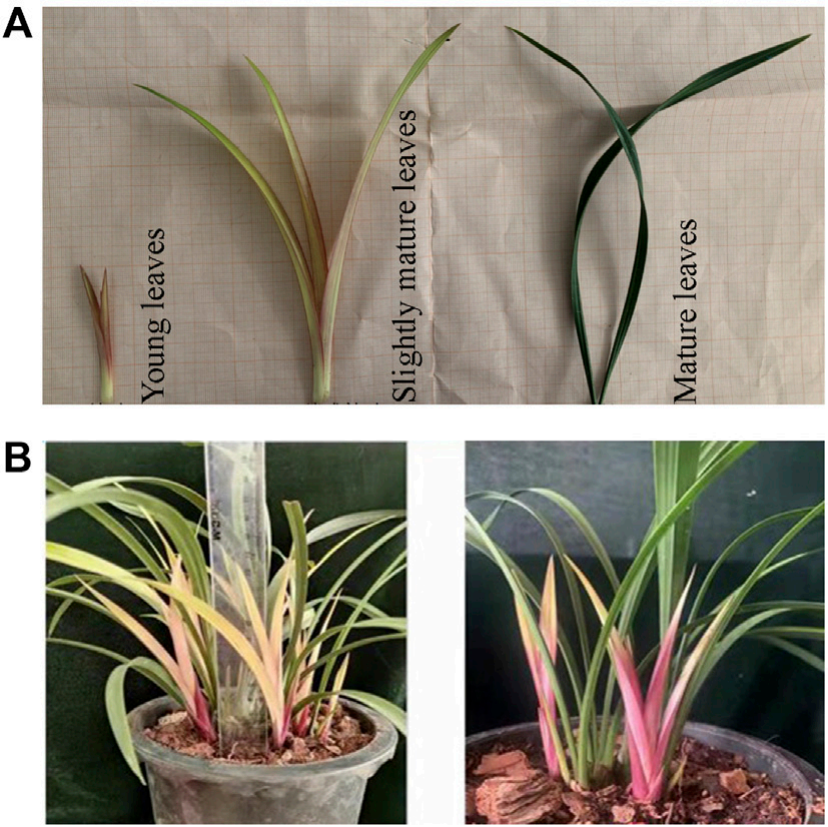
A pictorial description of *Cymbidium ensifolium* leaf coloration. **(A)** leaf color alteration pertaining to development. **(B)** leaf color variation in mature *C. ensifolium* leaves depicting yellow, purple, and green coloration.

*Cymbidium ensifolium* changes leaf color over the developmental stages. We observed the leaf color variation in young, slightly mature, and mature leaves. Young leaves are purple, turning yellow, and finally green (Figure 1). All three colors can be observed in the mature plant, as shown in Figure 1B. The distinct coloration pattern increases the aesthetic appeal of *C. ensifolium* as an additional ornamental characteristic aside from flowers.

To understand the molecular mechanisms underlying color variation in leaf, we collected leaf samples at each stage in three replicates for further downstream analysis, *viz*., metabolite characterization and transcriptome profiling.

### Chlorophyll accumulation

**FIGURE 2:**
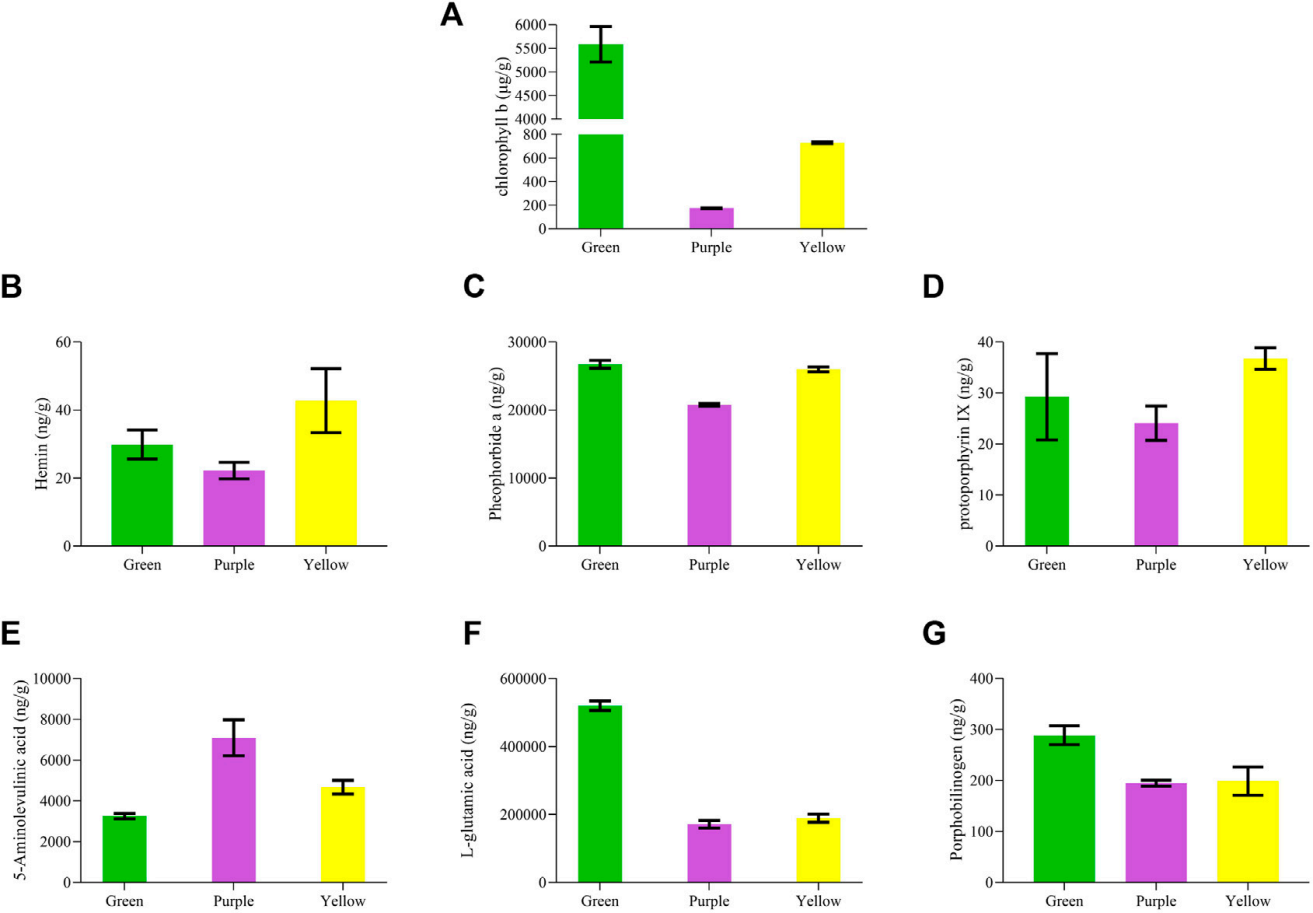
Chlorophyll profiling of purple, yellow, and green leaves in *C. ensifolium*. **(A)** chlorophyll b, **(B)** hemin, **(C)** pheophorbide, **(D)** protoporphyrin IX, **(E)** 5-aminolevulinic acid, **(F)** L-glutamic acid, and **(G)** porphobilinogen.

Chlorophyll accumulation was tested in three leaf tissues using LC-MS/MS analysis. Chlorophyll b contents were higher in green leaf tissue compared to yellow and purple (Figure 2A). Purple leaf tissues had the lowest accumulation of chlorophyll b contents. Both chlorophylls a and b were tested for the respective accumulation; however, we found that with a normal accumulation pattern of chlorophyll b, chlorophyll a was not detected. We assumed that the chlorophyll a standard product might be degraded. To verify this, the samples were compared with the 500 ppm mixed standard (YLS_500 ppm_N). The comparison results showed normal peaks for chlorophyll b, whereas no peaks were observed for chlorophyll a, conferring the degradation of chlorophyll a standard product (Supplementary Figure S1).

Moreover, we quantified several compounds, which are usually a by-product of chlorophyll degradation, such as porphobilinogen, hemin, pheophorbide a, protoporphyrin, 5-aminolevulinic acid, and L-glutamic acid (Figures 2B–G). Hemin depicted a significantly higher accumulation in yellow leaves than in purple and green. Pheophorbide a and protoporphyrin IX were estimated with higher accumulation in yellow leaves but lowest in purple leaves. Moreover, porphobilinogen and L-glutamic acid increased with the change in color from purple to yellow to green. By contrast, 5-aminolevulinic acid gradually decreased with the color variation from purple to yellow to green.

### Anthocyanin profiling

Leaf tissue samples were further studied using target metabolite identification (UPLC MS/MS) for anthocyanins, resulting in the identification of 40 anthocyanin compounds from five different subclasses, including cyanidin, peonidin, delphinidin, flavonoids, petunidin, pelargonidin, malvidin, and procyanidin (Supplementary Table S1). The quality was ensured by observing the instrument’s accuracy following quality control measures, as previously explained by Fiehn et al. (2000), Superimposed display analysis of mass spectrometry total ion current (TIC) and extracted ion chromatogram of samples were run at a different time (Supplementary Figure S2). The overlapped TIC suggested the stability of the instrument as a quality check.

**FIGURE 3:**
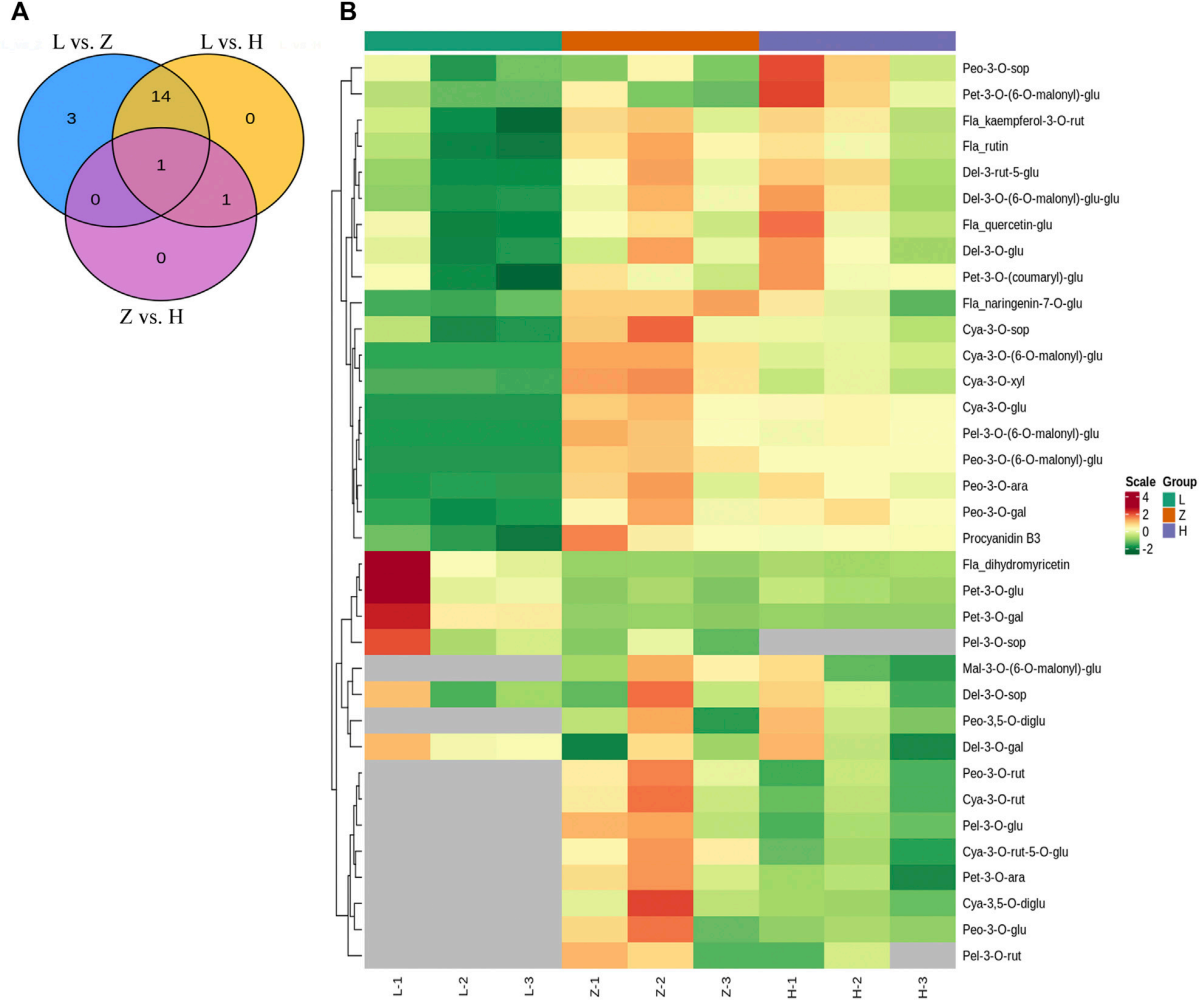
Anthocyanin profile. **(A)** venn diagram depicting differentially accumulated anthocyanins in each group with overlap, where L, H, and Z correspond to green, yellow, and purple leaves, respectively. **(B)** heatmap depicting accumulation pattern of identified anthocyanins in green, yellow, and purple leaf tissues. The numbers 1, 2, and 3 correspond to each replication.

Comparing green, yellow, and purple tissues yielded 16, 18, and 2 differentially accumulated anthocyanins in L vs. H, L vs. Z, and Z vs. H, respectively (Figure 3A). Delphinidin-3-O-glucoside, cyanidin-3-O-glucoside, cyanidin-3-O-(6-O-malonyl-beta-D-glucoside), cyanidin-3-O-xyloside, petunidin-3-O-(6-O-malonyl-beta-D-glucoside), pelargonidin-3-O-(6-O-malonyl-beta-D-glucoside), peonidin-3-O-galactoside, peonidin-3-O-arabinoside, peonidin-3-O-(6-O-malonyl-beta-D-glucoside), quercetin-3-O-glucoside, rutin, kaempferol-3-O-rutinoside, and delphinidin-3-O-(6-O-malonyl)-glucoside-3′-glucoside were identified with upaccumulation in yellow leaves compared to green leaves, suggesting a significant role of in differential coloration (Figure 3B and Supplementary Table S1). Although petunidin-3-O-galactoside, petunidin-3-O-glucoside, and dihydromyricetin depicted a down accumulation pattern in yellow leaves. The above-mentioned anthocyanins depicted a similar regulation pattern in comparison with L vs. Z, except for pelargonidin-3-O-(6-O-malonyl-beta-D-glucoside). Moreover, three anthocyanins, including cyanidin-3-O-sophoroside, naringenin-7-O-glucoside, and procyanidin B3, were upaccumulated explicitly in purple leaves compared to green leaves (Figure 3B). The results signify the potential role of cyanidin-3-O-sophoroside, naringenin-7-O-glucoside, and procyanidin B3 in purple color formation in *C. ensifolium.* In the Z vs. H comparison, only two differentially accumulated anthocyanins were identified, including cyanidin-3-O-xyloside (upaccumulated) and petunidin-3-O-(6-O-malonyl-beta-D-glucoside) (downaccumulated).

### Flavonoids associated with leaf coloration

**FIGURE 4:**
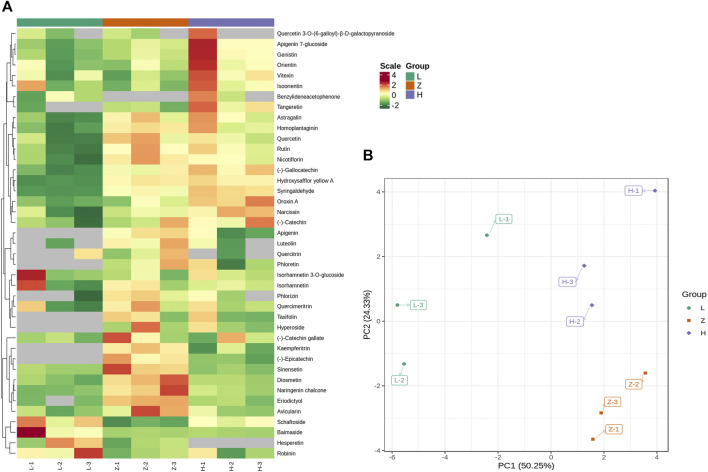
Differential accumulation of flavonoids in three leaf tissues, L (green leaves), H (Yellow leaves), and Z (purple leaves). **(A)** accumulation pattern of 41 DAFs (at least differentially expressed in one comparison). **(B)** PCA plot depicting PC-based distribution of samples and their replicates. L, H, and Z correspond to green, yellow, and purple leaves. The numbers 1, 2, and 3 correspond to each replication.

Targeted metabolic profile identified 67 flavonoids, including chalcones (6), flavanols (6), flavanones (7), flavone glycosides (4), flavones (18), flavonols (15), isoflavanones (3), phenolic acids (1), and other flavonoids (7) (Supplementary Table S2). PCA was performed to check the repeatability of data sets. The first two components, *viz*., PC1 and PC2, covered 74% variation and clustered replicates from each colored leaf (Figure 4B). We further compared samples from different colored leaf tissues and identified DAFs (Figure 4A). Comparison L vs. H identified 12 DAFs, including hydroxysafflor yellow A, tangeretin, oroxin A, syringaldehyde, nicotiflorin, rutin, genistin, astragalin, baimaside, apigenin 7-glucoside, homoplantaginin, and naringenin chalcone. All DAFs were upregulated in yellow leaves, whereas only one flavonoid homoplantaginin from the flavonoes subclass, depicted down accumulation in yellow leaves compared to green leaves (Supplementary Table S3). Likewise, a comparison of green leaves and purple leaves yielded 12 DAFs, including diosmetin, hydroxysafflor yellow A, tangeretin, syringaldehyde, nicotiflorin, rutin, schaftoside, astragalin, baimaside, sinensetin, homoplantaginin, naringenin, and chalcone. Except for rutin and baimaside, all DAFs depicted upaccumulation in purple leaves compared to green leaves (Supplementary Table S3). It is of interest that hydroxysafflor yellow A, tangeretin, syringaldehyde, nicotiflorin, rutin, astragalin, homoplantaginin, and naringenin chalcone were upaccumulated in both yellow and purple leaves compared to green leaves. Therefore, the color variation from purple to yellow to green can be attributed to the variable levels of these flavonoids.

Moreover, a comparison of purple and yellow tissues identified six DAFs (two upaccumulated and four downaccumulated). Tangeretin and schaftoside depicted upaccumulation in yellow leaves, whereas diosmetin, eriodictyol, sinensetin, and naringenin chalcone were downaccumulated in yellow leaves. Diosmetin, sinensetin, and naringenin chalcone were identified with upaccumulation in purple leaves compared to yellow and green leaves (Supplementary Table S3). The results emphasized that a gradual decrease in diosmetin, sinensetin, and naringenin chalcone accumulation might be the reason for differential coloration.

### Transcription control of leaf color variation

**FIGURE 5:**
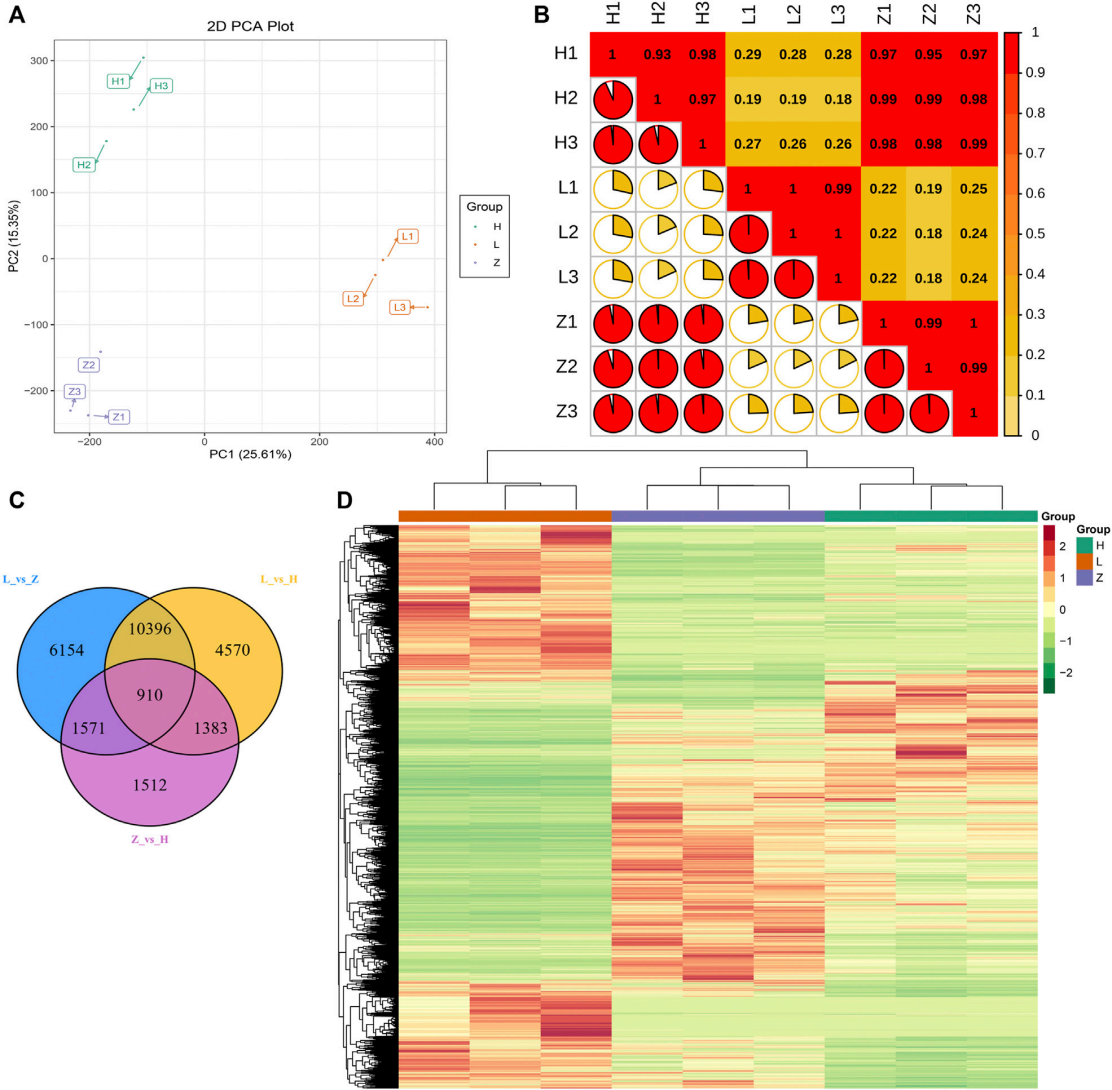
Transcriptome profiling of three different colored leaves of *C. ensifolium*. **(A)** PCA plot based on FPKM values of all the quantified genes. **(B)** correlation plot of quantified genes. **(C)** venn diagram representing differentially expressed genes and their overlap in comparison between green, yellow, and purple leaves, viz., L vs. H, L vs. Z, and Z vs. H. **(D)** heatmap representing FPKM values of all the quantified genes through transcriptome profiling.

The metabolome is referred to as the end product in biological pathways. Therefore, to identify the genes responsible for differential accumulation of anthocyanins, flavonoids, and chlorophyll, we sequenced the transcriptome of different colored leaf tissues. A total of nine cDNA libraries were constructed with three replicates for each color group. The transcriptomic sequencing yielded 0.44 billion raw reads, and after filtering low-quality reads, we obtained 0.43 billion clean reads with 64.71 Gb of data (Supplementary Table S4). After mapping, the FPKM values were calculated based on read counts and used for further downstream analysis. The quantified genes were subjected to PCA and PCC. The results signify the credibility and repeatability of transcriptomic datasets. The first two PCs covered 40.96% variation, and replicates from each colored group were clustered together (Figure 5A). PCC results also emphasized a strong correlation among the replicates from each colored group (Figure 5B).

The transcriptome datasets were further analyzed to identify DEGs related to color variation. Overall FPKM values for the genes differentiated the identified genes into three groups consistent with the leaf color (Figure 5D). Thus, we compared each group and further categorized DEGs from each group. A total of 26,499 DEGs (at least differentially expressed in one comparison) were identified (Supplementary Table S5). Comparison L vs. H depicted 17,259 DEGs with 9,236 downregulated and 8,023 upregulated genes in yellow leaves compared to green leaves. Likewise, comparisons L vs. Z and Z vs. H identified 19,031 DEGs (9,320 downregulated and 9,711 upregulated) and 5,376 DEGs (2,586 downregulated and 2,790 upregulated), respectively. The annotation information of identified DEGs in each group suggested enrichment of pathways related to photosynthesis, phenylpropanoid biosynthesis, flavonoid biosynthesis, and circadian rhythm (Supplementary Figures S3–S5). We further identified 910 conserved DEGs between the three groups (Figure 5C). A comparison of DEGs from each group suggested that more changes in gene expression are required for green coloration than yellow and purple. Moreover, we identified 151 DEGs (at least expressed in one comparison) related to flavonoid biosynthesis, isoflavonoid biosynthesis, phenylpropanoid biosynthesis, flavone, and flavonol biosynthesis.

**FIGURE 6:**
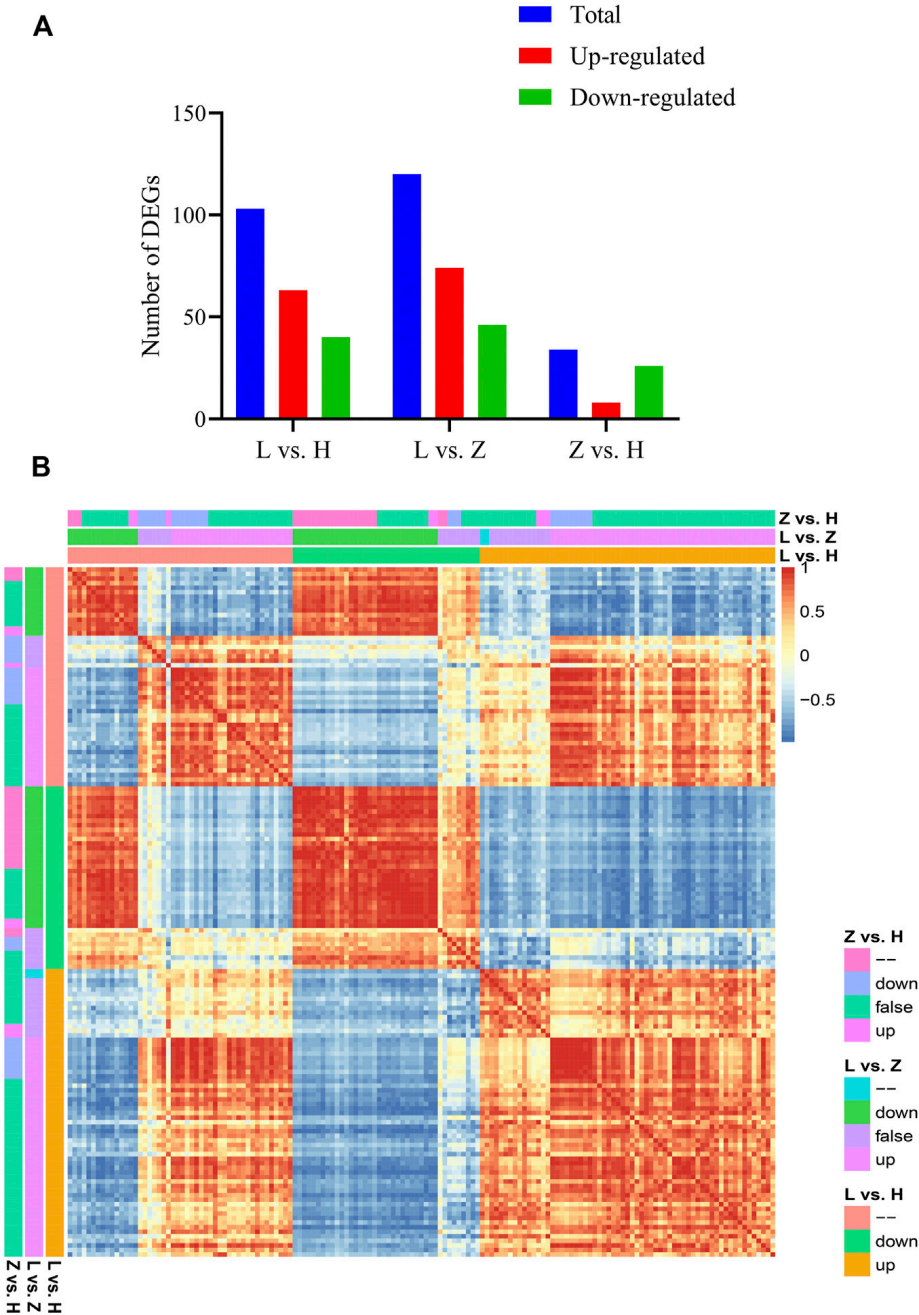
Characterization of color-related DEGs identified based on the annotation information. **(A)** statistics for color-related DEGs in three comparisons between green, yellow, and purple leaves, *viz*., L vs. H, L vs. Z, and Z vs. H. **(B)** correlation plot representing PCC between color-related DEGs (151), where down = downregulated, up = upregulated, false = log_2_Fold Change is between −1 and 1.

Based on annotation information, we further characterized the identified DEGs related to differential coloration. A total of 151 DEGs (at least differentially expressed in one comparison) were identified as color regulators. Differential statistics for these DEGs have been presented in Figure 6. In three comparisons, *viz*., L vs. H, L vs. Z, and Z vs. H, 103, 120, and 34 DEGs were identified, respectively (Figure 6A). It is interesting that leaf color variation from purple to yellow (Z vs. H) depicted the lowest number of overall DEGs and color-related DEGs, further emphasizing our assumption of minimal changes in gene expression from purple to yellow while requiring extensive changes in color variation from purple to yellow to green. We further characterized the expression of each gene in three tissues. Moreover, based on PCC calculated using corresponding FPKM values, the identified DEGs can be classified into five groups (Figure 6B).

Forty genes encoding cytochrome P450, UDP-glucoronosyl, and UDP-glucosyl transferase, PDDEXK-like, non-haem dioxygenase in morphine synthesis N-terminal, O-methyltransferase domain, glycosyltransferase 4-like domain, NAD-dependent epimerase/dehydratase family, 2OG-Fe (II) oxygenase superfamily, Kelch motif, heam oxygenase, and Myb-like DNA-binding domain depicted significant upregulation in green leaves compared to yellow and purple leaves, suggesting a significant role of these DEGs in regulating pigmentation in *C. ensifolium* leaves. On the other hand, 11 genes, *viz*., *Cluster-12991.59020*, *Cluster-12991.60709*, *Cluster-12991.120400*, *Cluster-12991.121138*, *Cluster-12991.121355*, *Cluster-12991.116930*, *Cluster-12991.92049*, *Cluster-12991.108016*, *Cluster-12991.110524*, *Cluster-12991.106370*, and *Cluster-12991.108125*, depicted conserved differential expression in all three comparisons. These genes encode cytochrome P450, NmrA-like family, O-methyltransferase domain, UDP-glucoronosyl, UDP-glucosyl transferase, chalcone and stilbene synthases, N-terminal domain, and AMP-binding enzyme. It is interesting that only cytochrome was upregulated in green leaves compared to yellow and purple leaves, whereas genes encoding NmrA-like family, O-methyltransferase domain, UDP-glucoronosyl, UDP-glucosyl transferase, chalcone, stilbene synthases N-terminal domain, and AMP-binding enzyme displayed the highest expression in purple leaves. Therefore, we assume that differential expression of these genes might be the key reason for leaf color variation in *C. ensifolium*, and a gradual decrease in expression leads to the color variation from purple to yellow to green leaves.

Moreover, 31 DEGs were upregulated in green leaves compared to yellow and purple leaves. These genes include cytochrome P450 CYP2 subfamily, iron/ascorbate family oxidoreductases, UDP-glucuronosyl and UDP-glucosyl transferase, flavonol reductase/cinnamoyl-CoA reductase, iron/ascorbate family oxidoreductases, FOG: Kelch repeat, heam oxygenase, Myb superfamily, and their homologs. On the other hand, only two genes, *viz*., *Cluster-12991.164172* and *Cluster-12991.59020* (cytochrome P450 homologs), Genes in green leaves are compared with yellow and purple leaves (Supplementary Table S5).

### Genes and pathways regulating leaf coloration in *C. ensifolium*

**FIGURE 7:**
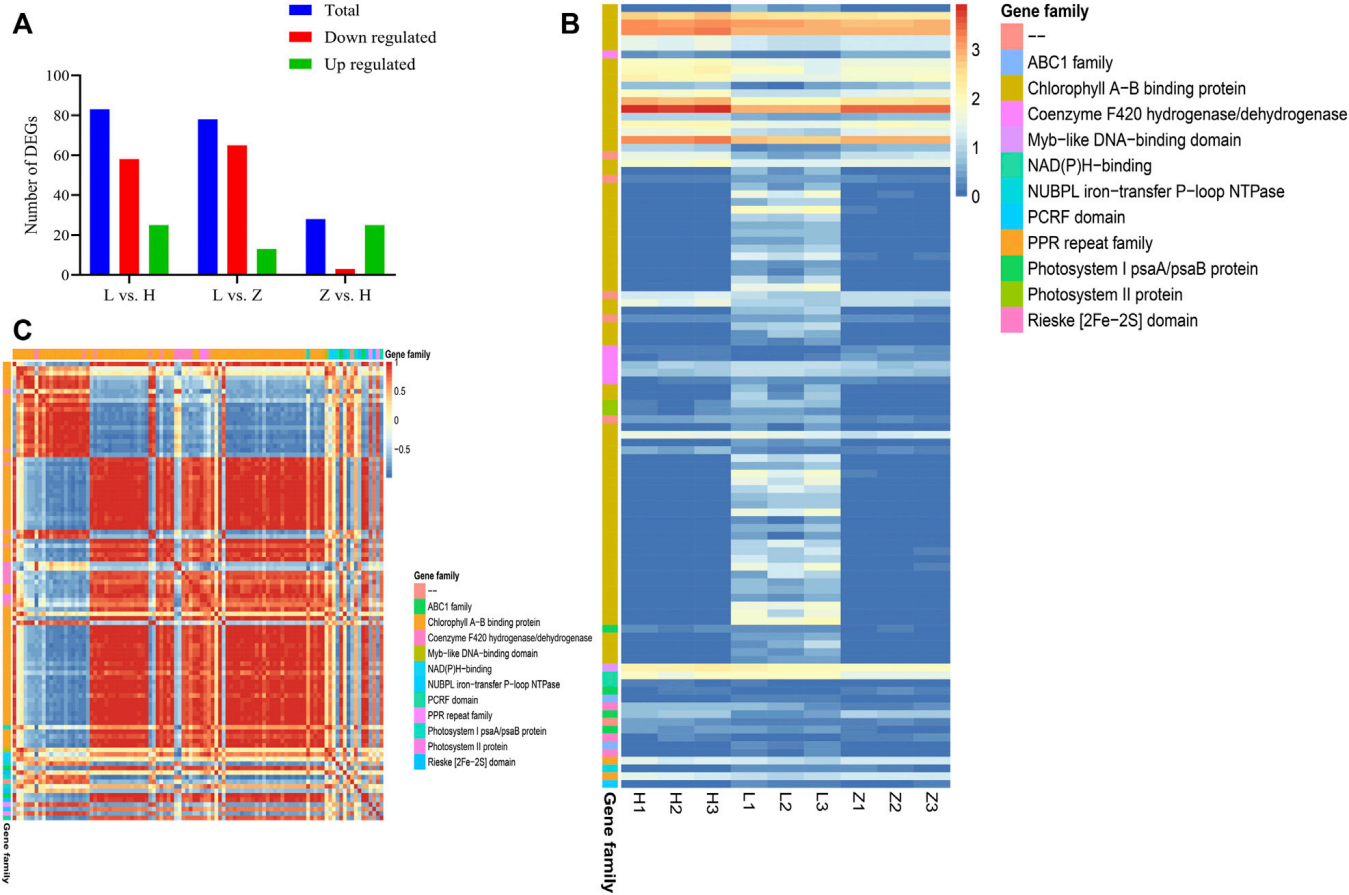
Characterization of DEGs associated with the chlorophyll contents. **(A)** statistics for chlorophyll-related DEGs in three comparisons between green, yellow, and purple leaves, *viz*., L vs. H, L vs. Z, and Z vs. H. **(B)** heatmap of 151 chlorophyll-related DEGs based on corresponding FPKM values. **(C)** correlation plot representing PCC between 101 chlorophyll-related DEGs.

Together, chlorophyll contents, anthocyanins and flavonoid profiling, and transcriptomic characterization improve our understanding of leaf color variation in *C. ensifolium*. Chlorophyll contents are major pigmentation for green color variation in plants. Therefore, we characterized the genes associated with chlorophyll contents and identified 101 DEGs associated with chlorophyll contents. These DEGs encoding chlorophyll A–B binding protein, coenzyme F420 hydrogenase/dehydrogenase, photosystem II protein, Myb-like DNA-binding domain, NAD(P)H-binding, photosystem I psaA/psaB protein, ABC1, Rieske (2Fe-2S) domain, PPR, NUBPL iron transfer P-loop NTPase, and PCRF were identified (Figure 7 and Supplementary Table S5). It is interesting that the number of DEGs increased from purple to yellow to green leaves, which is consistent with a gradual increase in chlorophyll contents. Comparison Z vs. H depicted upregulation of 25 genes, whereas 65 and 58 DEGs depicted upregulation in green leaves compared to purple and yellow leaves, respectively (Figures 7A,B). The results provide a molecular basis to further manipulate the chlorophyll accumulation pattern in *C. ensifolium*.

**FIGURE 8:**
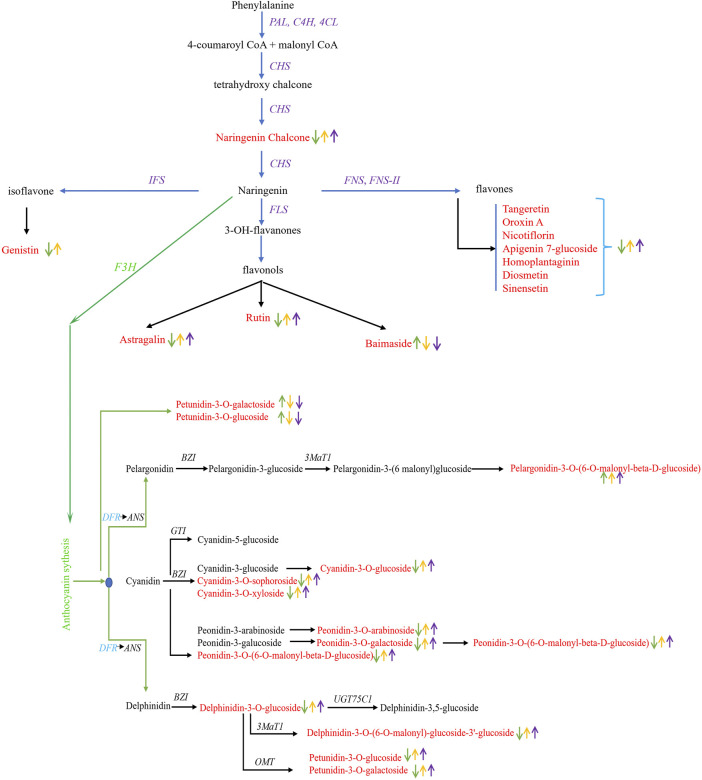
Differential regulation of flavonoid biosynthesis in *C. ensifolium* leaves. Differentially accumulated flavonoids and anthocyanins are presented in red text. The upward and downward arrows in green, yellow, and purple color indicated the accumulation pattern in green, yellow, and purple leaves, respectively. The pathway was reconstructed by following the layout and nomenclature available on https://www.genome.jp/kegg/kegg2.html.

Anthocyanins accumulation pattern in three colored leaves suggested the absence of cyanidin-3,5-O-diglucoside, pelargonidin-3-O-glucoside, peonidin-3,5-O-diglucoside, cyanidin-3-O-rutinoside, malvidin-3-O-(6-O-malonyl-beta-D-glucoside), peonidin-3-O-glucoside, peonidin-3-O-rutinoside, petunidin-3-O-arabinoside, cyanidin-3-rutinoside-5-glucoside, delphinidin-3-O-(6-O-malonyl-beta-D-glucoside), and pelargonidin-3-O-rutinoside in green leaves. Based on annotation information, we screened 22 DEGs from four gene families: transferase family, cytochrome P450, ABC1 family, and UDP-glucosyl transferase (Supplementary Table S5). It is interesting that most of the DEGs associated with anthocyanin accumulation were downregulated in green leaves, except for four genes, *viz*., *Cluster-12991.114567*, *Cluster-12991.113887*, *Cluster-12991.201069*, and *Cluster-12991.153858*. Moreover, dihydroflavonol 4-reductase (DFR), a major gene in the anthocyanin biosynthesis pathway, was identified with downregulation in green leaves compared to yellow and purple leaves. Seven genes, *viz*., *Cluster-12991.119374*, *Cluster-12991.119375*, *Cluster-12991.119368*, *Cluster-12991.119369*, *Cluster-12991.119371*, *Cluster-12991.119372*, and *Cluster-12991.119373* were identified encoding *DFR* protein (Supplementary Table S5). Anthocyanin accumulation pattern and differential regulation of genes encoding *DFR* suggested the key role of DFR genes in regulating leaf color in *C. ensifolium* (Figure 8). Together, the differential regulation of genes associated with anthocyanin accumulation regulates leaf coloration in *C. ensifolium*.

### qRT-PCR-based validation of selected genes

**FIGURE 9:**
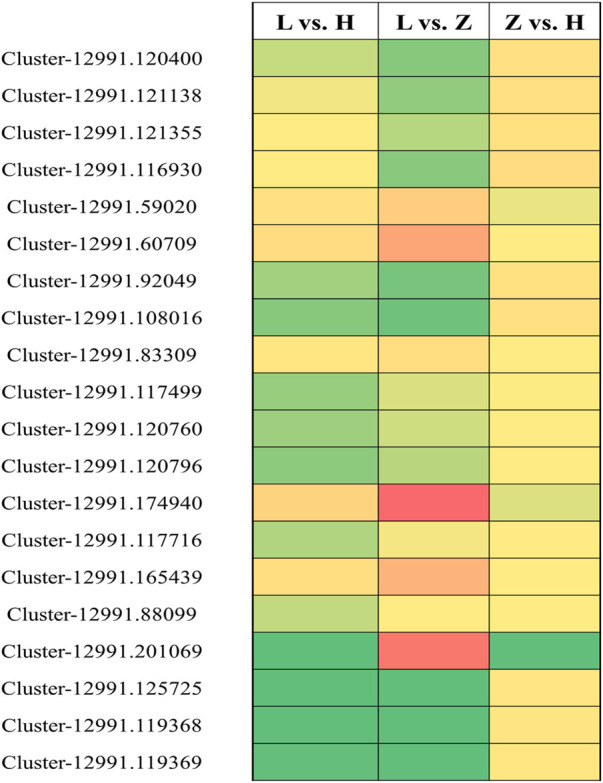
qRT-PCR-based verification for 20 selected genes related to flavonoid biosynthesis, chlorophyll accumulation, and anthocyanin synthesis. Green, yellow, and red boxes indicate unchanged, moderately upregulated, and highly upregulated genes, respectively.

We selected 20 genes based on their corresponding annotation information and differential expression in green, yellow, and purple leaves to further verify their expression pattern through qRT-PCR (Supplementary Table S6). Similar to expression patterns in transcriptome data, qRT_PCR results depicted significant variation in the regulation pattern of the 20 selected genes (Figure 9). Among the 20 genes, 9, 7, and 4 were related to flavonoid biosynthesis, chlorophyll accumulation, and anthocyanin biosynthesis, respectively. Comparisons L vs. H and L vs. Z identified the most significant variation in gene expression. *Cluster-12991.59020*, *Cluster-12991.60709*, *Cluster-12991.174940*, *Cluster-12991.165439*, and *Cluster-12991.201069* were identified with higher expression in purple tissues compared to yellow and green tissues. It is interesting that all the genes except *Cluster-12991.125725* showed similar expression patterns in yellow and purple leaves, whereas *Cluster-12991.125725* showed a lower expression level in yellow leaves than in purple leaves.

## Discussion

The leaf sheath color in *C. ensifolium* ranges from purple to yellow to green. Aside from flower coloration, leaf color variation is an important additional ornamental characteristic. Flavonoids are involved in the formation and development of flowers, fruits, and seeds in plants and other functions such as antioxidant activity, UV protection, and protection against biotic and abiotic stresses (Samanta et al., 2011; Baskar et al., 2018; Chin et al., 2018; Agati et al., 2020). Most studies concerning ornamentals generally focus on flower color variation and leaf shape (de Souza et al., 2012; Zhao and Tao, 2015; Manzoor et al., 2019). However, the molecular basis of *C. ensifolium* leaf color variation has not been studied. In this study, we systematically used metabolomics and transcriptomics approaches to identify the major contributors to leaf coloration and screened key genes associated with coloration, providing critical information for the enrichment of *C. ensifolium* germplasm.

Chlorophyll, flavonoids, and anthocyanins are the primary factors influencing the coloration of different plant organs (Guo et al., 2009; Li et al., 2019). Chlorophyll is the primary pigment developing green color while varying concentration of carotenoids and anthocyanins is necessary for plants’ yellow to purple pigmentation (Wilkinson et al., 2002; Croft et al., 2017). Our results suggested significant differences in chlorophyll accumulation in purple, yellow, and green leaves. In general, chlorophyll degradation coupled with anthocyanins and carotenoids leads to leaf color variation from green to yellow to reddish. However, in *C. ensifolium,* leaf color changes from purple to yellow to green during developmental phases. Therefore, an increase in chlorophyll contents with successive development provides a strong basis for color variation in *C. ensifolium.* Moreover, the chlorophyll-related DEGs also depicted significantly higher expression in green leaves, signifying the role of chlorophyll accumulation in green pigmentation.

Several studies have demonstrated the essential role of anthocyanins in color formation. For instance, Xue et al. (2021) depicted the association of anthocyanins with red-colored seed coats in peanuts. A study by Qiu et al. (2020) concerning passion fruit found that the total anthocyanin contents of purple fruit were significantly higher than that of yellow fruit. White, yellow, blue, and pink *Primula vulgaris* (Li et al., 2020) showed a gradual increase in total anthocyanin content as the color deepened. We identified several anthocyanins with differential expressions in purple, yellow and green leaves. It is interesting that cyanidin-3-O-sophoroside, naringenin-7-O-glucoside, and procyanidin B3 were identified as upaccumulated in purple leaves. Several studies have reported that variable concentrations of cyanidin-3-O-sophoroside result in the development of purple pigmentations in plants (Terahara et al., 2004; Morita et al., 2005; Oliveira et al., 2006). Moreover, Yin et al. (2019) identified naringenin-7-O-glucoside as a potential contributor to the reddish petal color in *Brassica napus*. Moreover, copigmentation resulting from the differential accumulation of delphinidin, cyanidin, petunidin, and quercetin contributed to yellow leaves, and these results are in line with the findings of several studies (Nielsen et al., 2005; Lambert et al., 2011; Benmeziane et al., 2016; Fu et al., 2018). Several key genes encoding NmrA-like family, O-methyltransferase domain, UDP-glucoronosyl, UDP-glucosyl transferase, chalcone synthesis (CHS), and stilbene synthases N-terminal domain and AMP-binding enzyme were identified as color regulators. CHS is a key regulator in anthocyanin accumulation pathways (Yang et al., 2002). Yang et al. (2002) characterized the CHS gene family as a contributor to the petal color variation in Dendranthema. Further functional verification of these identified genes can unveil regulatory mechanisms underlying pigmentation in *C. ensifolium.*

Likewise, differential accumulation patterns of flavonoids, such as diosmetin, sinensetin, and naringenin chalcone, depicted a gradual decrease from purple to yellow to green leaves. We considered these flavonoids to be key leaf coloration regulators based on annotation information and previously published statistics. Diosmetin has been previously identified as a major contributor to indigo pigmentation in woad (Pauk et al., 2022). By contrast, sinensetin, along with rosmarinic acid and eupatorine, were identified in *Orthosiphon aristatus*, regulating color variation from purple to white (Faramayuda et al., 2020). Several DEGs were identified related to phenylpropanoid pathways, including UDP-glucoronosyl and UDP-glucosyl transferase, flavodoxin, cytochrome P450, AMP-binding enzyme, multicopper oxidase, Kelch motif, aldehyde dehydrogenase family, and Myb-like DNA-binding domain.

Several metabolites and key genes were identified through transcriptome and metabolome analysis. Transcriptome analysis is an important tool for identifying genes responsible for a stage-specific trait. The analysis of different comparison groups revealed that only a few genes showed an upregulation trend in expression as leaf color deepened, suggesting that the process of color deepening requires only a few key involvements. Moreover, the functional enrichment of DEGs indicated that changes in differential gene expression caused significant changes in metabolic activities. The results suggested that copigmentation of several anthocyanins and flavonoids governed by structural genes resulted in the color change of *C. ensifolium* leaves. The study provides a molecular basis for leaf coloration in *C. ensifolium*. However, further functional verification of identified genes can provide insights into regulatory pathways underlying pigmentation in *C. ensifolium*. Moreover, *C. ensifolium* leaves can be used as a source of natural pigmentation.

## Data Availability

Data used in this study is available as accession number PRJNA790719 in the public database of the National Center of Biotechnology Information.
