# Supplementary figures and images for: Insight into the molecular mechanisms of leaf coloration in Cymbidium ensifolium
Source: Front Genet. 2022 Aug 12;13:923082. doi: 10.3389/fgene.2022.923082 (PMC9413228; doi:10.3389/fgene.2022.923082)

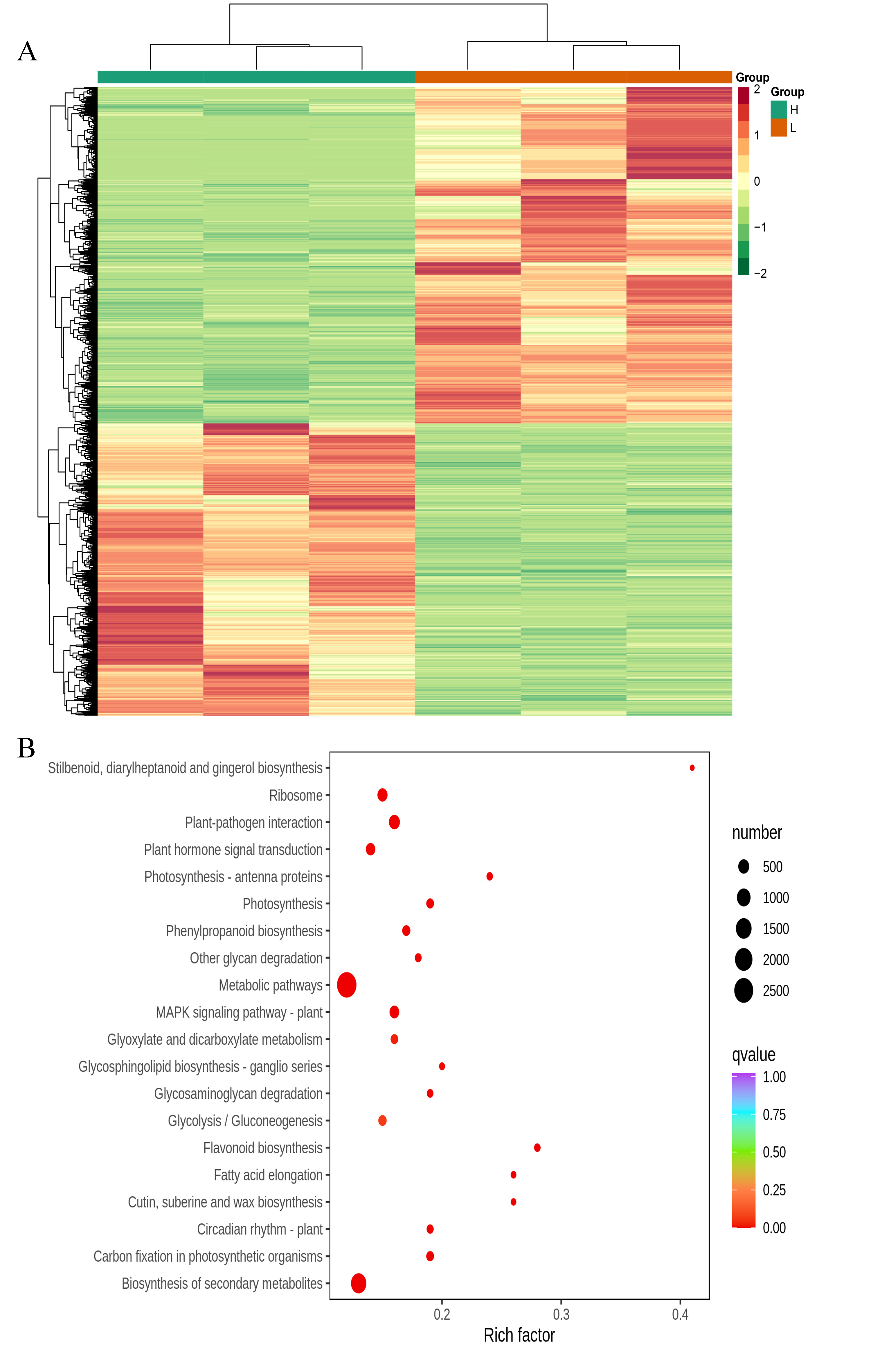

Supplement: Supplementary file 2 [file Image3.JPEG]

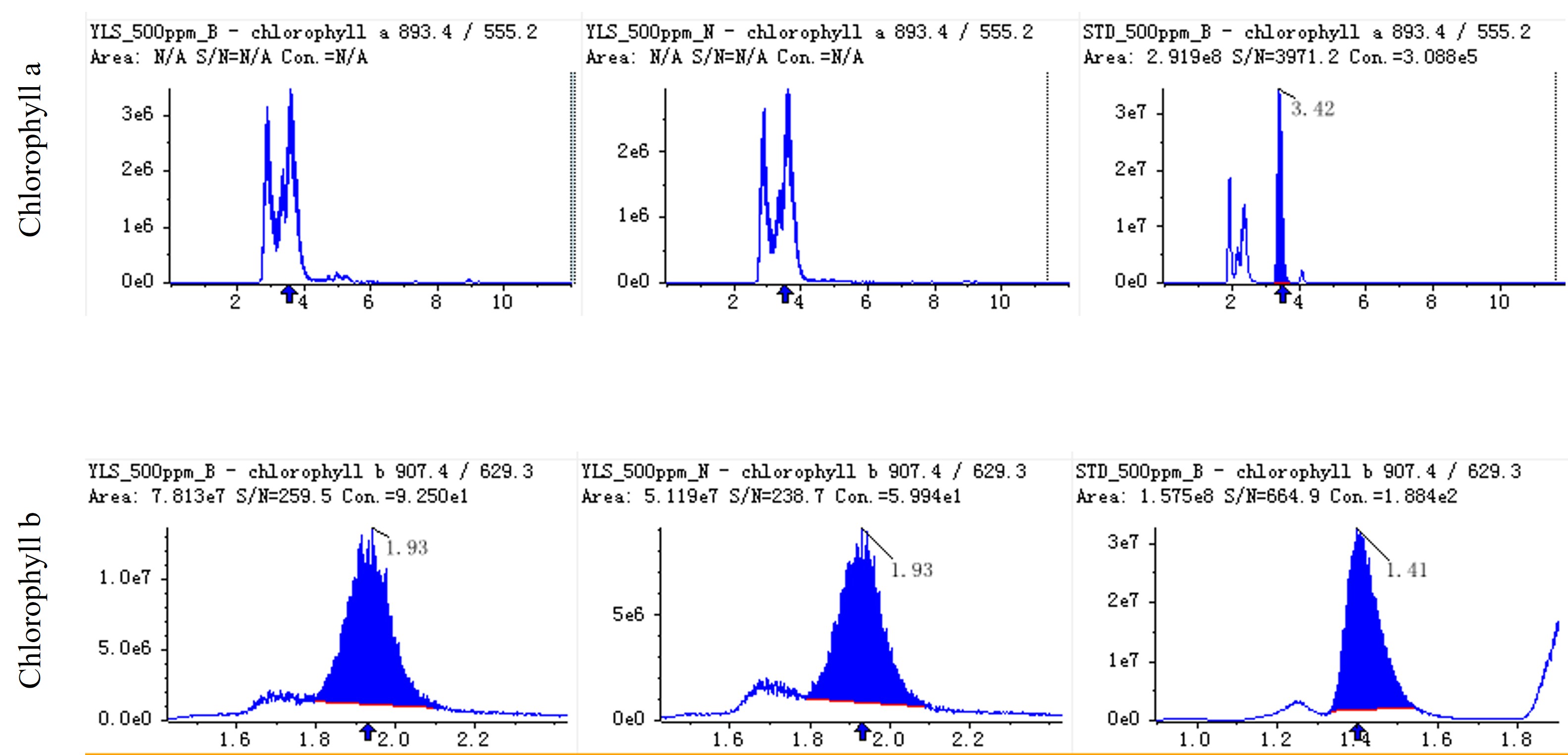

Supplement: Supplementary file 4 [file Image1.JPEG]

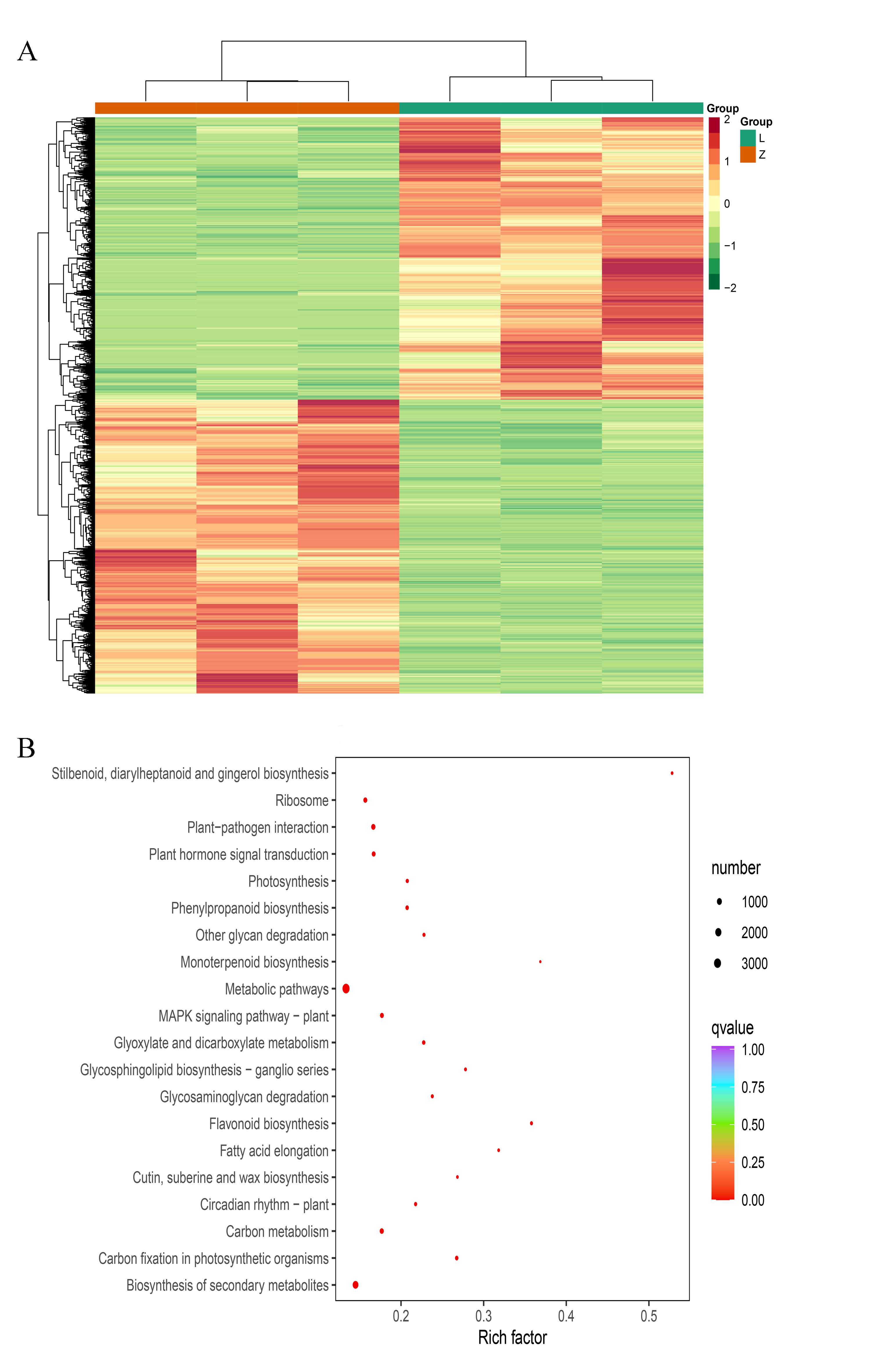

Supplement: Supplementary file 5 [file Image4.JPEG]

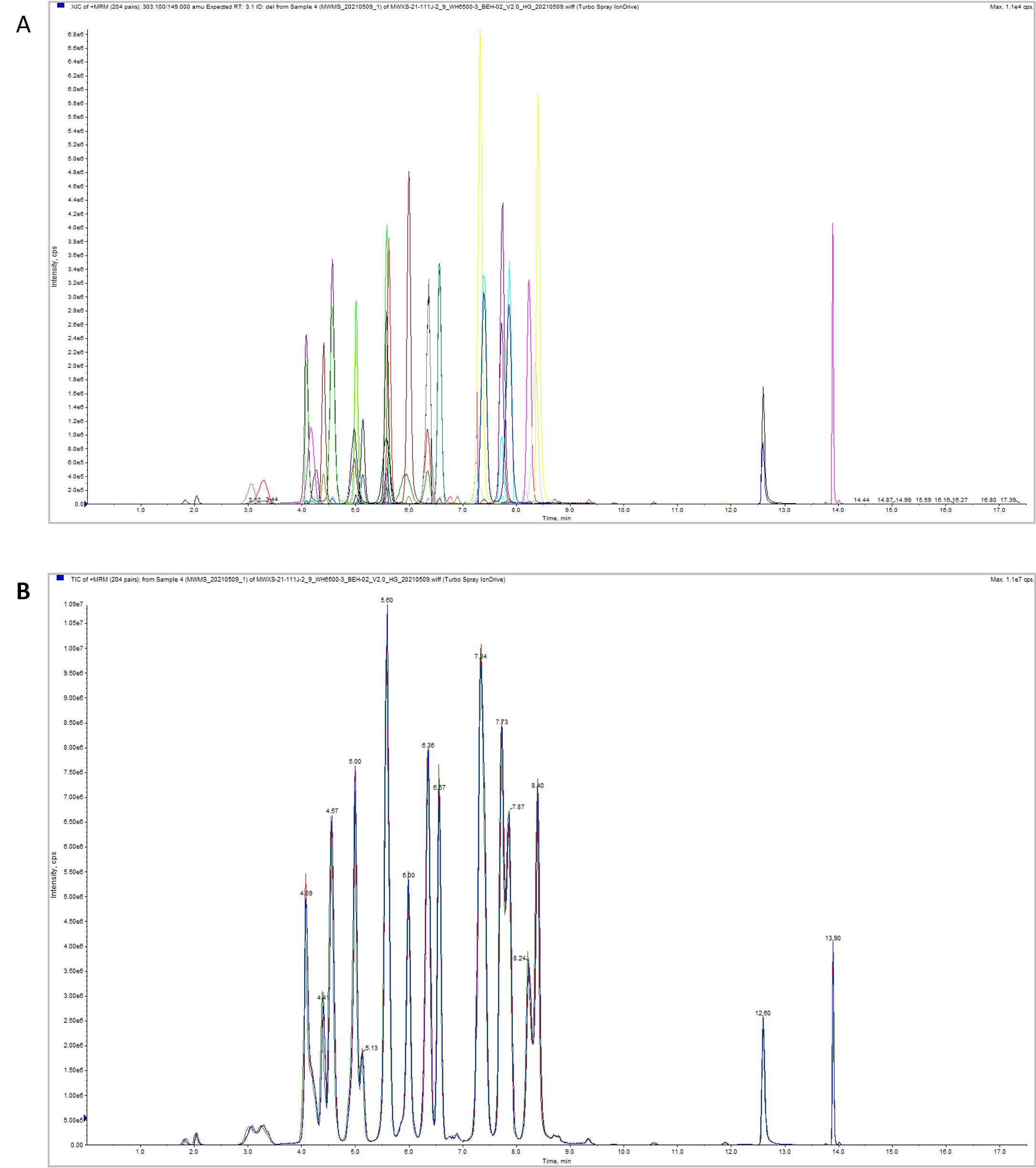

Supplement: Supplementary file 6 [file Image2.JPEG]

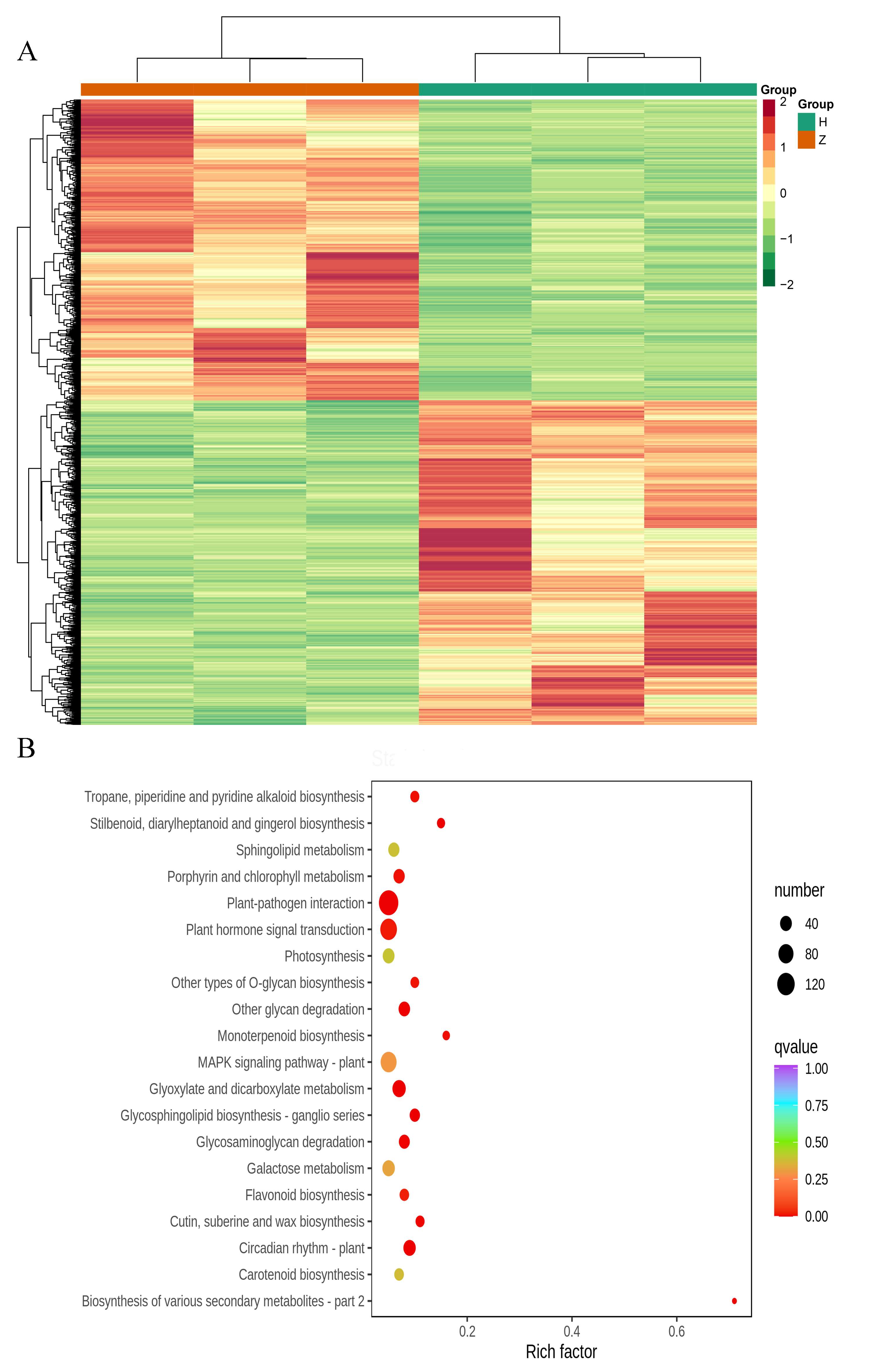

Supplement: Supplementary file 7 [file Image5.JPEG]
